# Healthcare utilization is a collider: an introduction to collider bias in EHR data reuse

**DOI:** 10.1093/jamia/ocad013

**Published:** 2023-02-08

**Authors:** Nicole G Weiskopf, David A Dorr, Christie Jackson, Harold P Lehmann, Caroline A Thompson

**Affiliations:** Department of Medical Informatics and Clinical Epidemiology, Oregon Health & Science University, Portland, Oregon, USA; Department of Medical Informatics and Clinical Epidemiology, Oregon Health & Science University, Portland, Oregon, USA; Department of Medical Informatics and Clinical Epidemiology, Oregon Health & Science University, Portland, Oregon, USA; Division of Health Science Informatics, Department of Medicine, Johns Hopkins University School of Medicine, Baltimore, Maryland, USA; Department of Epidemiology, Gillings School of Global Public Health, University of North Carolina at Chapel Hill, Chapel Hill, North Carolina, USA; Division of Cancer Epidemiology, Lineberger Comprehensive Cancer Center, University of North Carolina at Chapel Hill, Chapel Hill, North Carolina, USA

**Keywords:** collider bias, EHR data reuse, real-world data, cohort selection, directed acyclic graphs

## Abstract

**Objectives:**

Collider bias is a common threat to internal validity in clinical research but is rarely mentioned in informatics education or literature. Conditioning on a collider, which is a variable that is the shared causal descendant of an exposure and outcome, may result in spurious associations between the exposure and outcome. Our objective is to introduce readers to collider bias and its corollaries in the retrospective analysis of electronic health record (EHR) data.

**Target audience:**

Collider bias is likely to arise in the reuse of EHR data, due to data-generating mechanisms and the nature of healthcare access and utilization in the United States. Therefore, this tutorial is aimed at informaticians and other EHR data consumers without a background in epidemiological methods or causal inference.

**Scope:**

We focus specifically on problems that may arise from conditioning on forms of healthcare utilization, a common collider that is an implicit selection criterion when one reuses EHR data. Directed acyclic graphs (DAGs) are introduced as a tool for identifying potential sources of bias during study design and planning. References for additional resources on causal inference and DAG construction are provided.

## INTRODUCTION

One of the most promising applications of electronic health record (EHR) data is in the potential to harness these data to generate knowledge and evidence that is representative of and generalizable to target patients. Much has been written about the limited generalizability of clinical trials and other prospective studies that may target participants who differ significantly from the populations of interest in terms of demographics or clinical profiles.^1^ EHR-based research may serve as a more generalizable compliment to prospective research paradigms. These data provide an opportunity to learn about population health, the natural history of disease, and the impact of system-level and individual interventions.

EHR data are not, however, a random sample of the population in the United States. Multiple studies have shown, for example, that sicker patients have more data documented in the EHR, meaning that inclusion criteria that require a certain quantity of documented and accessible data may bias a study sample toward sicker patients.^2–4^ Characteristics of the healthcare system also impact the availability of data: data are not shared across many healthcare settings, leading to data that are more representative of the type of providers or healthcare capabilities than of the health of the patient.^5^^,^^6^ Alternatively, care fragmentation leads to duplicative efforts with resulting poor data quality.^7^ Finally, there are community- and patient-level factors that impact access to care (and, consequentially, inclusion in the EHR population), including financial security, transportation capabilities, distance to care, and a number of other social determinants of health.^8–10^

The complex mechanisms surrounding access to care and healthcare utilization will be reflected in EHR data, but the typical EHR-based analysis does not take into account potential biases resulting from these complexities. Such analyses would benefit from an improved understanding of the challenges and potential negative outcomes of reusing these data without properly considering and acting upon these data generation mechanisms. Specifically, there is often a failure to acknowledge that reuse of clinical data may result not only in poor generalizability (failure of external validity) when the patient sample does not match the target population, but in spurious associations (failure of internal validity), even when the intent is not to determine causation. (The distinction between external and internal validity is discussed in more detail below.)

The use of causal models (represented with directed acyclic graphs, or DAGs, as described below) makes apparent a common source of spurious associations that arises due to selecting or conditioning on a *collider*, which is a variable that is a causal descendant of 2 or more other variables. If, for example, you want to test for a relationship between 2 variables of interest—a risk factor and an outcome—but both of those factors precipitate (or cause) a third variable, which the third variable is a collider. Conditioning on that collider may occur due either to sample selection or to adjusting in analyses. In both cases, conditioning on a collider (or descendent of a collider) can inadvertently introduce selection bias that may falsely reverse, exaggerate, induce, or eliminate the true relationship between a risk factor and outcome of interest, even when properly adjusted for confounding. In other words, not all adjusting is good, and care is needed to be sure you are doing the proper adjustment.

Collider-stratification bias is a common threat to the validity of retrospective studies, including those that rely on EHR data. In fact, one of the most well-known forms of collider bias, Berkson’s bias, arises when sampling is conditioned upon hospital admission. In a paper published in 1946, Berkson demonstrated that 2-by-2 tables comparing the incidence of 2 conditions within an inpatient setting showed an association that was not present in the general population.^11^ Moreover, the ongoing Coronavirus disease 2019 (COVID-19) pandemic has highlighted the dangers of conducting retrospective biomedical research without sufficient awareness of potential colliders.^12^ Nevertheless, as of September of 2022, the terms “collider” and “collider bias” are almost entirely absent from informatics literature (Supplementary Appendix). The current article, which draws heavily upon epidemiologic concepts of causality and bias, is intended as an introduction to the concept of collider bias and its corollaries in the retrospective analysis of EHR data. While colliders are an unavoidable result of data-generating processes and can take many forms, we focus specifically on problems that may arise from conditioning on forms of healthcare utilization, which is an implicit selection criterion when one reuses EHR data.

## DIRECTED ACYCLIC GRAPHS IN EPIDEMIOLOGY

We use DAGs throughout this article to illustrate assumed or known causal relationships between variables and to demonstrate potential biases. In epidemiology, DAGs are a common and powerful tool for guiding retrospective research design and analyses. DAGs consist of nodes representing variables and directed edges representing known, assumed, or hypothesized causal paths. All DAGs must include an exposure (or risk factor) and outcome. Other variables with causal relationships to the exposure, outcome, or both may also be included. It should be noted that the knowledge and assumptions encoded in DAGs are not unique to causal inference; researchers should always consider these factors when designing a study. DAGs are merely a standardized visual representation of this information that are intended to make hypotheses and assumptions explicit, as well as to aid with the identification of potential biases.

While different DAG conventions exist, we have chosen to employ those used by DAGitty, which is tool for creating and understanding DAGs (DAGitty can be run from a browser, downloaded, or used via an R package).^13^ We represent exposures as green-shaded nodes and outcomes as blue-shaded nodes. Other variables are grey if the model does not adjust (control) for them and white if it does. When the edges in the graph are colored pink this indicates that bias may exist along that pathway.

It is important to reiterate that DAGs are knowledge-derived. While they can be tested empirically, they are meant to be constructed prior to beginning a study or analysis, and serve as a tool to codify known or theorized relationships in order to identify potential sources of bias beforehand. The encoding of knowledge in DAGs is what makes them so powerful for guiding study design and avoiding bias, but also means that DAG construction must be learned and practiced. As in any study, identifying relevant variables and determining their causal relationships to the exposure and outcome may require consultation with experts, literature review, or both. A complete introduction to DAG construction is beyond the scope of this article, and we have provided references for a number of excellent introductions and tutorials on the topic.^14–19^ Additionally, the DAGitty website includes tutorials, primers, and additional resources on this topic (http://www.dagitty.net/).

## A COLLIDER BIAS EXAMPLE

Consider the following example. A class of medical students is asked to determine if there is an association between 2 conditions with related symptoms, but no definitive biological connection: gout and rheumatoid arthritis (there is debate^20^^,^^21^ around whether these 2 conditions are associated, but in our toy example we assume that no association exists). The students are provided with access to the complete medical charts of 10 000 randomly selected adult patients receiving care at their institution.

Half the students extract the relevant data from all 10 000 charts. The others decide to limit their sample to those patients seen at the medical center’s rheumatology clinic, assuming they will capture the relevant population more efficiently. The 2 groups employ a comparable data extraction approach, using a combination of data queries and chart review to determine whether each patient has gout, rheumatoid arthritis, neither, or both.

At the end of the term, the students report their results. The group that included all 10 000 patients finds that there is no association between gout and rheumatoid arthritis, with a risk ratio of 1.0. The group that limited their sample to rheumatology patients, however, estimates a negative association between the 2 conditions (risk ratio of <1.0); they conclude that gout might be protective against rheumatoid arthritis. Both groups used the same statistical approach, so why were their results different?

**Figure 1.**
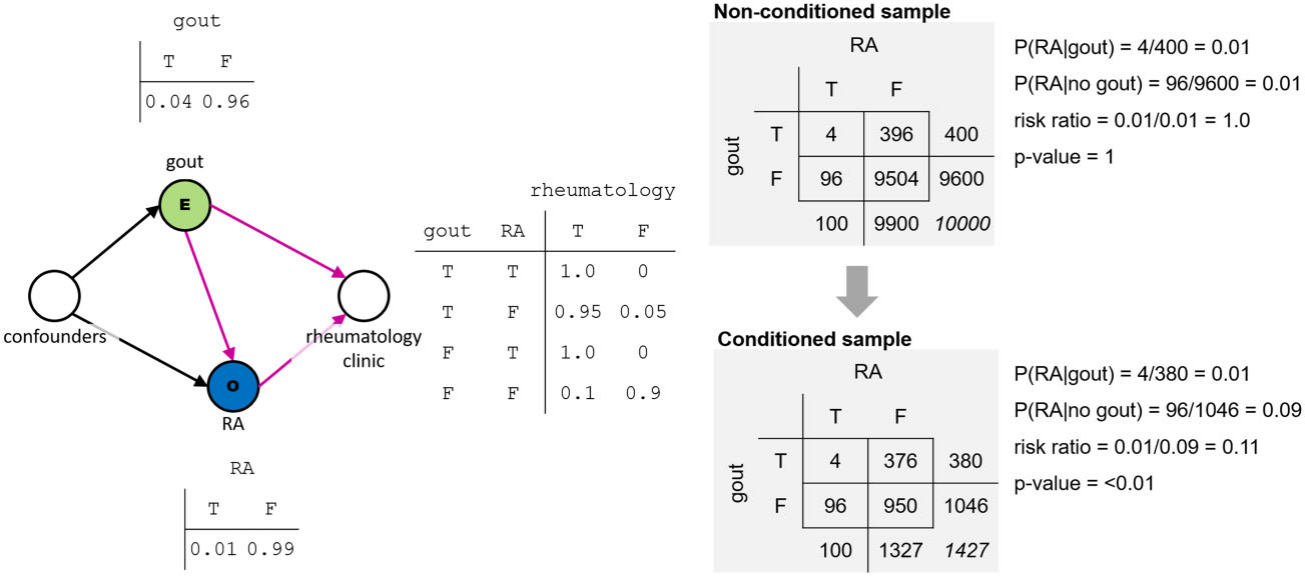
In our example, we assume that gout (exposure) and RA (outcome) are unrelated. A random sample of all patients will show that no relationship exists (top right). If, however, we condition our selection on a collider (eg, coming to rheumatology clinic), as shown in this DAG, we introduce bias. The probability of being selected for the sample is equivalent to the probability of receiving rheumatology care, which is higher for patients with gout or RA than for patients with neither. The resulting sample will show a significant association between the 2 conditions, even though, at the population level, there is no relationship (bottom right). DAG: directed acyclic graph.

The difference occurs because the second group of students conditioned their sampling on a collider: receiving care through the rheumatology clinic. Individuals with gout or rheumatoid arthritis have an increased likelihood of being a patient of the rheumatology clinic, meaning that both diagnoses are drivers of this third variable. This study design is depicted in a DAG in Figure 1. A directed edge points from gout (our hypothesized risk factor) to rheumatoid arthritis (our hypothesized outcome), indicating a hypothesized causal path (eg, gout causes rheumatoid arthritis). Because we assume that both conditions change the probability of a patient receiving care at a rheumatology clinic (ie, each is an independent causal factor), there are directed edges pointing from each condition to the rheumatology clinic node. Finally, while we do not focus on confounders in this article, for clarity we have chosen to represent them in all DAGs and assume they are measured correctly and adequately adjusted. As noted above, the identification of confounders and other relevant variables (mediators, modifiers, and other covariates) is important.

The resulting selection bias is clear if we consider the 2 groups’ data. The data from the full sample are summarized in the top right of Figure 1. Based on these data and the calculated risk ratio of 1.0, we conclude (as intended) that gout and rheumatoid arthritis are independent from one another. The data limited to the rheumatology clinic (bottom right of Figure 1), however, include only 1427 of the total 10 000 patients. The key difference is that this sampling was biased: patients were far more likely to be included if at least one of the 2 factors of interest (gout or rheumatoid arthritis) were present, since patients with these conditions are more likely to receive specialty rheumatology care than patients with neither condition. And especially so if they were the rare patient to have both. As a result, the patients with neither condition are underrepresented in the limited sample, leading to the observation of a spurious association between the 2 conditions, with an observed risk ratio of 0.11, markedly lower than the true value of 1.0. Had this second group of students collected data on 1427 *randomly* selected patients, rather than those receiving rheumatology care, it is unlikely that they would have observed this association between gout and rheumatoid arthritis.

**Figure 2.**
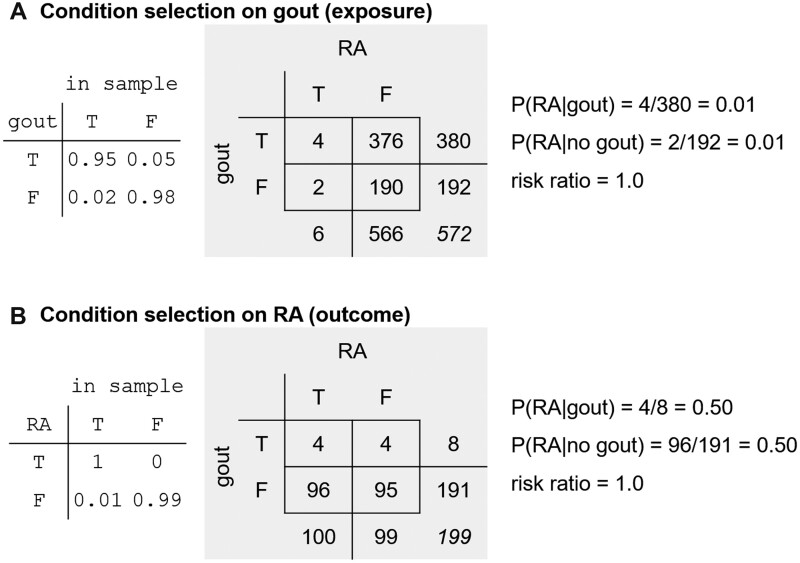
Conditioning selection on an exposure (panel A) or an outcome (panel B) may impact conditional probabilities (in this case, the probability of having RA given the presence of gout), but will not impact risk ratios and other ratio-based metrics.

It is important to note that conditioning on only the outcome (having rheumatoid arthritis, in this example) may result in incorrect estimates of conditional probabilities, but not in the *ratio* of the 2 conditional probabilities (ie, odds ratio). Imagine that each month there is a special clinic devoted to rheumatoid arthritis, which is utilized by all patients with confirmed or suspected rheumatoid arthritis. The data collected from patients seen in this clinic are shown in Figure 2, panel B. While the conditional probability of having rheumatoid arthritis given the presence of gout is elevated (0.50 vs 0.01) with this selection method, the *relative risk* of having rheumatoid arthritis given the presence or absence of gout is the same as if all 10 000 cases were considered (this is akin to case–control sampling, in which cases are intentionally oversampled, which distorts estimates of absolute risk, but provides useful estimates of relative risk).^22^ If we were to consider the opposite scenario (selection is conditioned on having gout), the relative risk is once again the correct 1.0 (Figure 2A).

## A NOTE ON SELECTION BIAS VERSUS GENERALIZABILITY

Students in informatics and related disciplines are often taught that a finding is internally valid if what is observed is true within the study, and externally valid if those findings can also be generalized to a population of interest (internal validity is often considered to be a necessary prequisite for external validity^23^). While these definitions generally suffice for randomized controlled trials, in retrospective studies the distinction becomes more nuanced. Although one could argue that the spurious associations between gout and rheumatoid arthritis described above are not subject to the commonly-considered forms of research error (ie, there is no measurement error or uncontrolled confounding and the statistics were run correctly), this spurious association would still be considered a problem with internal validity, since the finding is due to bias arising from selecting based on a collider variable. It may be helpful to think about this scenario at the patient-level, rather than in aggregate: based on the results described in the scenario above, would you advise differing testing standards for patients with gout versus those without? No, because the data do not indicate an actual protective effect of gout against rheumatoid arthritis; therefore, what we have observed is a threat to the internal validity of this analysis.

## COROLLARIES

In addition to the example above, which focuses on collider bias that arises from conditioning on healthcare utilization that is driven by 2 clinical concepts, there are additional ways in which utilization-related colliders may lead to bias without appropriate consideration. The first occurs when selection is conditioned on a *descendant* of healthcare utilization, which may be less obvious than when selection is conditioned on utilization itself. The second occurs when EHR data are used to examine the association between a clinical outcome and a non-clinical exposure that may drive healthcare access (ie, social determinants of health).

### Conditioning on a descendant of a collider

**Figure 3.**
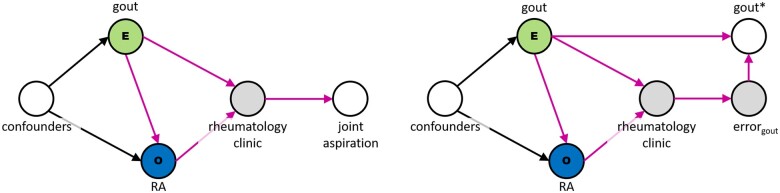
Conditioning or selecting on the descendant of a collider (rheumatology clinic) also introduces bias. In the left panel, selection is conditioned on joint aspiration results, the gold standard for gout diagnosis, which is most often performed by a rheumatologist. In the right panel, selection is conditioned on presence of a gout diagnosis, which is more likely to be present for a patient who has received specialty rheumatology care.

As mentioned above, conditioning on the descendant of a collider of the exposure and outcome will induce bias in the same manner as conditioning on the collider itself. For example, imagine that the researchers do not limit their sample to patients who have been to the rheumatology clinic, but they do require that a gout diagnosis be confirmed by joint aspiration, a procedure which is more commonly performed by rheumatologists than primary care providers. Conditioning on the presence of joint aspiration results, therefore, will mostly limit the sample to patients who have received care through the rheumatology clinic, leading to the same erroneous observation of a protective effect of gout against rheumatoid arthritis (Figure 3, left).

### Selection into an EHR-derived cohort

The majority of EHR data reuse scenarios begin with the definition of a patient cohort. Cohorts are often defined through the presence of one or more key clinical variables within different time frames and can be unexpectedly complex to operationalize and implement.^24^^,^^25^ EHR data quality plays a role in cohort identification, because the data (often diagnosis codes) used to determine inclusion in a cohort may be incorrectly present or, more often, incorrectly absent.^26–28^ Non-random data quality problems, typically referred to as information bias,^29^ provide another opportunity for unintentionally conditioning on a collider, resulting in selection bias.

Consider again our question of whether gout is a risk factor for rheumatoid arthritis. Patients with gout are identified via the presence of a relevant diagnostic code. We can refer to the presence of this code as observed gout, which is an imperfect proxy for true gout status. (In reality, the same would be true for rheumatoid arthritis, but for the sake of simplicity we are choosing to assume that rheumatoid arthritis status is observed without error in this example.) We know diagnosis codes are prone to some degree of measurement error. The question, then, is whether this error occurs at random or is driven by an underlying mechanism that could be endogenous to our study question data generating mechanism. As shown in Figure 3 in the second panel, there is reason to believe that patients seen by a rheumatology specialist are more likely to have rheumatologic diagnoses properly documented in their records. Therefore, the error mechanism underlying observed gout is in fact a descendent of the rheumatology clinic collider. If we revisit our DAG from Figure 1, we can represent both the measurement error and the fact that this error may be differential when comparing patients who do, and do not utilize rheumatology care.

### Social determinants of health and healthcare utilization

**Figure 4.**
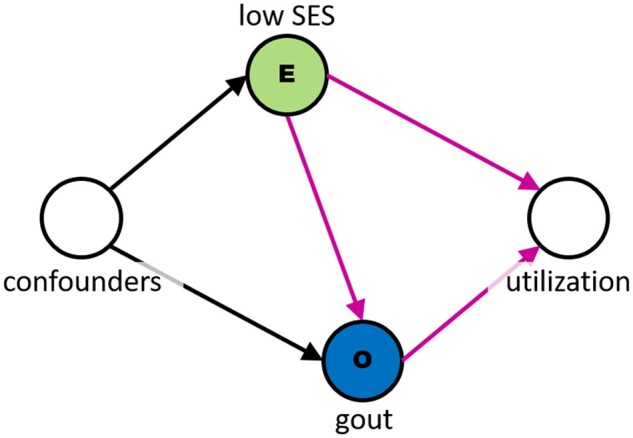
Healthcare utilization is a collider for low socioeconomic status (SES), which impacts access to healthcare, and gout status, which drives need for healthcare.

While our previous example focused on patients who received rheumatology care, healthcare utilization (ie, the amount and type of healthcare services in which a patient engages) can take many forms, including primary, emergent, specialty, and inpatient care. Healthcare utilization is a necessary precursor to having a record present in an EHR and, in general, is driven not only by clinical factors but also by access to healthcare and associated social determinants of health. It is therefore important to consider what would happen if the “risk factor” of interest is a social determinant of health. It has been hypothesized, for example, that low socioeconomic status is a risk factor for gout,^30^ but socioeconomic status is also a driver of healthcare utilization. Looking at Figure 4, it is clear that an EHR-derived patient sample used to determine if there is an association between low socioeconomic status and gout will by definition be subject to selection bias due to conditioning on a utilization, which is a collider. The observed odds ratio of having gout with low versus high socioeconomic status will be higher than the true relative risk that would be observed in a random sample of the overall population.

## AVOIDING BIAS FROM CONDITIONING ON COLLIDERS

While some types of collider stratification bias can be mitigated or adjusted in *post hoc* analyses (see Hernan et al.,^31^ and Nohr and Liew^32^), in this article we have focused mainly on the most egregious type of collider bias, which occurs when one conditions upon a common child of the exposure and outcome of interest (Figure 1), or upon a descendant of that common child (Figure 3). It is important to note that this type of collider bias cannot be fixed with *post hoc* adjustment and is a fatal error if it is implicit in the study design, that is, if the study subject selection process resulted in this conditioning.

It is an inescapable truth of EHR-based retrospective research that subject selection will always, by definition, be dependent upon healthcare utilization; patients who have not utilized healthcare will not have a chart, and therefore cannot be included in a study sample. Moreover, the vast majority of clinical research questions that one might ask of these data will include an exposure and outcome that are both drivers of healthcare utilization, at least in the United States. How, then, is it possible to avoid conditioning on colliders when conducting EHR-based clinical research?

The short answer is that when using EHR data to conduct clinical research, it is generally not possible to avoid collider bias. However, it may be possible to minimize this bias through careful cohort selection. Different forms of healthcare utilization (eg, primary care, specialty care, inpatient services, etc.) may more or less strongly driven by different exposures and outcomes. In our primary example, we have intentionally chosen to condition selection on the form of utilization most likely to result in bias: specialty rheumatology care. The presence of gout or rheumatoid arthritis *substantially* changes the probability that a patient will receive specialty rheumatology care. Primary care, however, is less heavily influenced by the presence or absence of these 2 conditions, and therefore would be a better form of utilization to condition on. (It is true that in the United States, the probability of receiving primary care is influenced by presence of disease,^33^ so this would still be a collider—but likely one with less impact on these specific findings.) Additionally, when EHR-based findings are reported, colliders should be identified and the directionality of potential collider bias considered.

In order to entirely avoid conditioning on a healthcare utilization collider, one must use data that are not subject to the same utilization-driven selection processes as data from the majority of United States healthcare system. That is, we need a patient sample that is not driven by potential exposures and outcomes. Countries with robust universal healthcare and/or interoperable health record systems will produce less biased patient samples. In the United States, bias associated with healthcare utilization might be decreased, though not avoided, by utilizing data from healthcare systems that provide comprehensive primary and specialty care regardless of social determinants of health (similarly, true population-based registries can provided less biased samples).^34^ Hypothetically, studies performed on all-payer claims data or data from closed systems, such as Kaiser Permanente or the Veterans’ Affairs healthcare system, might be less subject to collider stratification bias than those relying on data from academic medical centers.

Further work is needed to characterize the degree and directionality of bias that arises when conditioning on a utilization collider. EHR-derived data provide an opportunity to conduct empirical research on this phenomenon. For example, it would be fairly straightforward to determine if estimates of relative risk (or similar metrics) for a hypothesized causal relationship vary depending upon the form of utilization. And, if so, to what extent? This type of data-driven interrogation of potential colliders, which is analogous to sensitivity analysis, may be valuable for developing broad, general principles, or for specific studies.

Though we focus in this article on utilization, it is important to be aware that colliders are a natural part of data generating mechanisms and may take many forms. Some colliders, unlike healthcare utilization, can be managed by avoiding selecting, conditioning, or stratifying on them, or their direct descendants. It is therefore of paramount importance to know which variables are colliders by drawing a DAG for each research question. For a more in-depth tutorial on DAGs and how to use them to reduce confounding bias and prevent collider stratification bias, we recommend Shrier and Platt,^16^ Williamson et al.,^14^ or Suttorp et al.^17^ It is important to note that purely data-driven variable selection processes will not differentiate between collider and confounder variables, so DAG knowledge, especially whether a variable is a collider, is also important to build valid statistical and machine-learning models.

Finally, we strongly advise partnering with epidemiologists when designing EHR-based studies, especially those that could be subject to intractable collider stratification bias.

## CONCLUSIONS

While a variety of methodological approaches exist to minimize the impact of bias from arising selection or stratification on a collider, the priority must be careful consideration of study design and potential structural relationships between variables of interest, or underlying data generating mechanisms. DAGs are a powerful tool for diagramming these relationships and identifying potential sources of bias, but researchers must employ a knowledge-driven approach in creating these diagrams *before* selection of data or analyses are conducted.

Any research or analytical project that relies upon EHR-derived data conditions participant selection upon healthcare utilization. The DAG of almost any question asked of EHR data, therefore, should include at least one form of utilization. The vital question, then, is whether the variables examined are both drivers of utilization and, if so, how to avoid introducing bias related to conditioning on this collider.

We encourage consumers of EHR-derived data to learn more about colliders and the epidemiologic approaches underlying these considerations, carefully consider data-generating mechanism, and partner with epidemiologists with relevant methodological expertise.

## Supplementary Material

ocad013_Supplementary_Data

## Data Availability

No data were analyzed for this article.
