# Supplementary material for: Healthcare utilization is a collider: an introduction to collider bias in EHR data reuse
Source: J Am Med Inform Assoc. 2023 Feb 8;30(5):971–7. doi: 10.1093/jamia/ocad013 (PMC10114115; doi:10.1093/jamia/ocad013)
Supplement: ocad013_Supplementary_Data [file ocad013_supplementary_data.docx]

# **Appendix**

**Search terms**: selection bias, sampling bias, collider bias, collider stratification bias, ascertainment bias, Berkson’s paradox

**Conducted**: 9/23/2022

| Heath Informatics Journal | Collider bias: 0  Sampling bias: 3  Selection bias: 2  Collider stratification bias: 0  Ascertainment bias: 0  Berkson’s paradox: 0 |
| --- | --- |
| BMC Medical Informatics and Decision Making | Collider bias: 1  Sampling bias: 12  Selection bias: 11  Collider stratification bias: 0  Ascertainment bias: 1  Berkson’s paradox: 0 |
| International Journal of Medical Informatics | Collider bias: 0  Sampling bias: 6  Selection bias: 6  Collider stratification bias: 0  Ascertainment bias: 1  Berkson’s paradox: 0 |
| Journal of Biomedical Informatics | Collider bias: 2  [Sampling bias](https://pubmed.ncbi.nlm.nih.gov/?term=%28%22Journal+of+Biomedical+Informatics%22%5BJournal%5D%29+AND+%28sampling+bias%29): 12  [Selection bias](https://pubmed.ncbi.nlm.nih.gov/?term=%28%22Journal+of+Biomedical+Informatics%22%5BJournal%5D%29+AND+%28selection+bias%29): 10  Collider stratification bias: 0  Ascertainment bias: 0  Berkson’s paradox: 0 |
| Journal of Innovation in Health Informatics | Collider bias: 0  [Sampling bias](https://pubmed.ncbi.nlm.nih.gov/?term=%28%22Journal+of+Biomedical+Informatics%22%5BJournal%5D%29+AND+%28sampling+bias%29): 0  [Selection bias](https://pubmed.ncbi.nlm.nih.gov/?term=%28%22Journal+of+Biomedical+Informatics%22%5BJournal%5D%29+AND+%28selection+bias%29): 0  Collider stratification bias: 0  Ascertainment bias: 0  Berkson’s paradox: 0 |
| IEEE Journal of Biomedical and Health Informatics | Collider bias: 0  [Sampling bias](https://pubmed.ncbi.nlm.nih.gov/?term=%28%22IEEE+Journal+of+Biomedical+and+Health+Informatics%22%5BJournal%5D%29+AND+%28sampling+bias%29): 3  [Selection bias](https://pubmed.ncbi.nlm.nih.gov/?term=%28%22IEEE+Journal+of+Biomedical+and+Health+Informatics%22%5BJournal%5D%29+AND+%28selection+bias%29): 2  Collider stratification bias: 0  Ascertainment bias: 0  Berkson’s paradox: 0 |
| Journal of Medical Internet Research | Collider bias: 0  [Sampling bias](https://pubmed.ncbi.nlm.nih.gov/?term=%28%22Journal+of+Medical+Internet+Research%22%5BJournal%5D%29+AND+%28sampling+bias%29): 54  [Selection bias](https://pubmed.ncbi.nlm.nih.gov/?term=%28%22Journal+of+Medical+Internet+Research%22%5BJournal%5D%29+AND+%28selection+bias%29): 44  Collider stratification bias: 0  [Ascertainment](https://pubmed.ncbi.nlm.nih.gov/?term=%28%22Journal+of+Medical+Internet+Research%22%5BJournal%5D%29+AND+%28ascertainment+bias%29) bias: 6  Berkson’s paradox: 0 |
| Journal of Medical Internet Research: Medical Informatics | Collider bias: 0  [Sampling bias](https://pubmed.ncbi.nlm.nih.gov/33688846/): 5  Selection bias: 3  Collider stratification bias: 0  Ascertainment bias: 0  Berkson’s paradox: 0 |
| Journal of Medical Systems | Collider bias: 0  [Sampling bias](https://pubmed.ncbi.nlm.nih.gov/30604101/): 1  Selection bias: 1  Collider stratification bias: 0  Ascertainment bias: 1  Berkson’s paradox: 0 |
| Healthcare Informatics Research | Collider bias: 0  [Sampling bias](https://pubmed.ncbi.nlm.nih.gov/30109154/): 1  Selection bias: 1  Collider stratification bias: 0  Ascertainment bias: 0  Berkson’s paradox: 0 |
| Studies in Health Technology and Informatics | Collider bias: 0  [Sampling bias](https://pubmed.ncbi.nlm.nih.gov/?term=%28%22Studies+in+Health+Technology+and+Informatics%22%5BJournal%5D%29+AND+%28sampling+bias%29): 15  [Selection bias](https://pubmed.ncbi.nlm.nih.gov/?term=%28%22Studies+in+Health+Technology+and+Informatics%22%5BJournal%5D%29+AND+%28selection+bias%29): 12  Collider stratification bias: 0  [Ascertainment](https://pubmed.ncbi.nlm.nih.gov/?term=%28%22Studies+in+Health+Technology+and+Informatics%22%5BJournal%5D%29+AND+%28ascertainment+bias%29) bias: 2  Berkson’s paradox: 0 |
| Journal of the American Medical Association | Collider bias: 0  [Sampling bias](https://pubmed.ncbi.nlm.nih.gov/?term=%28%22Studies+in+Health+Technology+and+Informatics%22%5BJournal%5D%29+AND+%28sampling+bias%29): 0  [Selection bias](https://pubmed.ncbi.nlm.nih.gov/?term=%28%22Studies+in+Health+Technology+and+Informatics%22%5BJournal%5D%29+AND+%28selection+bias%29): 10  Collider stratification bias: 0  [Ascertainment](https://pubmed.ncbi.nlm.nih.gov/?term=%28%22Studies+in+Health+Technology+and+Informatics%22%5BJournal%5D%29+AND+%28ascertainment+bias%29) bias: 0  Berkson’s paradox: 0 |
